# Fructooligosaccharides production by immobilized *Pichia pastoris* cells expressing *Schedonorus arundinaceus* sucrose:sucrose 1-fructosyltransferase

**DOI:** 10.1093/jimb/kuab036

**Published:** 2021-06-17

**Authors:** Enrique R Pérez, Duniesky Martínez, Carmen Menéndez, Dubiel Alfonso, Iván Rodríguez, Luis E Trujillo, Alina Sobrino, Ricardo Ramírez, Eulogio Pimentel, Lázaro Hernández

**Affiliations:** Departamento de Investigación-Desarrollo, Centro de Ingeniería Genética y Biotecnología de Sancti Spíritus (CIGBSS), Circunvalante Norte S/N, Olivos 3, Apartado Postal 83, Sancti Spíritus 60200, Cuba; Departamento de Investigación-Desarrollo, Centro de Ingeniería Genética y Biotecnología de Sancti Spíritus (CIGBSS), Circunvalante Norte S/N, Olivos 3, Apartado Postal 83, Sancti Spíritus 60200, Cuba; Grupo Tecnología de Enzimas, Dirección de Investigaciones Agropecuarias, Centro de Ingeniería Genética y Biotecnología (CIGB), Ave 31 entre 158 y 190, Apartado Postal 6162, Habana 10600, Cuba; Grupo Tecnología de Enzimas, Dirección de Investigaciones Agropecuarias, Centro de Ingeniería Genética y Biotecnología (CIGB), Ave 31 entre 158 y 190, Apartado Postal 6162, Habana 10600, Cuba; Departamento de Ingeniería Química, Facultad de Química-Farmacia, Universidad Central “Marta Abreu” de Las Villas, Carretera a Camajuaní Km. 5 y 1/2, Santa Clara, Villa Clara 50100, Cuba; Grupo Tecnología de Enzimas, Dirección de Investigaciones Agropecuarias, Centro de Ingeniería Genética y Biotecnología (CIGB), Ave 31 entre 158 y 190, Apartado Postal 6162, Habana 10600, Cuba; Departamento de Investigación-Desarrollo, Centro de Ingeniería Genética y Biotecnología de Sancti Spíritus (CIGBSS), Circunvalante Norte S/N, Olivos 3, Apartado Postal 83, Sancti Spíritus 60200, Cuba; Grupo Tecnología de Enzimas, Dirección de Investigaciones Agropecuarias, Centro de Ingeniería Genética y Biotecnología (CIGB), Ave 31 entre 158 y 190, Apartado Postal 6162, Habana 10600, Cuba; Grupo Tecnología de Enzimas, Dirección de Investigaciones Agropecuarias, Centro de Ingeniería Genética y Biotecnología (CIGB), Ave 31 entre 158 y 190, Apartado Postal 6162, Habana 10600, Cuba; Grupo Tecnología de Enzimas, Dirección de Investigaciones Agropecuarias, Centro de Ingeniería Genética y Biotecnología (CIGB), Ave 31 entre 158 y 190, Apartado Postal 6162, Habana 10600, Cuba

**Keywords:** Calcium alginate, Cell immobilization, Fructooligosaccharides, Fructosyltransferase, Pichia pastoris

## Abstract

Fructooligosaccharides (FOSs)—fructose-based oligosaccharides—are typical prebiotics with health-promoting effects in humans and animals. The trisaccharide 1-kestotriose is the most attractive inulin-type FOS. We previously reported a recombinant sucrose:sucrose 1-fructosyltransferase (1-SST, EC 2.4.1.99) from *Schedonorus arundinaceus* (*Sa*) that efficiently converts sucrose into 1-kestotriose. In this study, *Pichia pastoris* PGFT6x-308 constitutively expressing nine copies of the *Sa1-SST* gene displayed fructosyltransferase activity in undisrupted biomass (49.8 U/ml) and culture supernatant (120.7 U/ml) in fed-batch fermentation (72 hr) with sugarcane molasses. Toluene permeabilization increased 2.3-fold the *Sa*1-SSTrec activity of whole cells entrapped in calcium-alginate beads. The reaction with refined or raw sugar (600 g/l) yielded 1-kestotriose and 1,1-kestotetraose in a ratio of 8:2 with their sum representing above 55% (wt/wt) of total carbohydrates. The FOSs yield decreased to 45% (wt/wt) when sugarcane syrup and molasses were used as cheaper sucrose sources. The beads retained 80% residual *Sa*1-SSTrec activity after a 30-day batchwise operation with refined cane sugar at 30°C and pH 5.5. The immobilized biocatalyst is attractive for the continuous production of short-chain FOSs, most particularly 1-kestotriose.

## Introduction

Fructooligosaccharides (FOSs)—fructose-based oligosacchari-des—are prebiotic fibbers widely utilized as functional food ingredients and in the pharmaceutical industry. FOSs ingestion improves the composition of the resident intestinal microbiota by stimulating selectively the growth of probiotics (bifidobacteria and lactobacilli), which results in the competitive exclusion of pathogens. Other associated benefits are the reduction of serum cholesterol, the increase in calcium and magnesium absorption, the prevention of colon cancer, and the production of B-vitamins. Additionally, FOSs are noncariogenic, low-calorie sugars of sweet taste and exhibit antioxidant activity by free radical scavenging (Cunha et al., 2019; Faria et al., 2021; Kaplan & Hutkins, 2000; Maiorano et al., 2020; Pereira & Gibson, 2002; Perna et al., 2018).

Commercially available FOSs contain a terminal α-d-glucose residue (G) and two to four fructosyl units (F) linearly connected by β-(2→1) linkages. Among inulin-type FOSs, the trisaccharide 1-kestotriose (1-kestose, GF_2_) has a superior bifidus-stimulating effect and tastes sweeter than the tetrasaccharide 1,1-kestotetraose (nystose, GF_3_) and the pentasaccharide 1,1,1-kestopentaose (fructosyl-nystose, GF_4_) (Mendlik et al., 2012; Suzuki et al., 2006). With these attributes, 1-kestotriose when supplemented with a more intense low-calorie sweetener (e.g., stevia) is adequate for replacing table sugar in prebiotic diabetic recipes that reduce the risk of obesity (Mabel et al., 2008).

Current industrial systems for inulin-type FOSs production are based on the use of β-fructofuranosidases (EC 3.2.1.26) and β-fructosyltransferases (EC 2.4.1.9) from few species of filamentous fungi, mainly from the genera *Aspergillus* and *Aureobasidium*. The reaction needs to be conducted at high sucrose concentrations (600–700 g/l) to restrict substrate hydrolysis. Under this condition, the fungal enzymes produce a mixture of GF_2_, GF_3_, and GF_4_, with their sum representing around 55% (wt/wt) of total carbohydrates (Cunha et al., 2019; Fernandez et al., 2007; Sánchez-Martínez et al., 2020).

FOS-synthesizing enzymes of different origins have been produced in transgenic yeasts (Hernández et al., 2018; Rehm et al., 1998; Trujillo et al., 2001; Yang et al., 2016). Our group reported a recombinant sucrose:sucrose 1-fructosyltransferase (1-SST, EC 2.4.1.99) from the plant *Schedonorus arundinaceus* (*Sa*) with catalytic properties highly attractive for the production of short-chain inulin-type FOSs (Hernández et al., 2018). The enzyme produced in *Pichia pastoris* (*Sa*1-SSTrec) yielded 1-kestotriose and 1,1-kestotetraose in an invariable ratio of 9:1 and overall FOSs content above 55% (wt/wt) from sucrose at the wide concentration range of 100–800 g/l. Fructose release to water (sucrose hydrolysis) was negligible even at the lowest substrate concentration (Hernández et al., 2018). The *P. pastoris* multicopy clone PGFT6x-308 secreted *Sa*1-SSTrec as a glycoprotein into the periplasmic space (38%) and the extracellular medium (62%) in fed-batch fermentation using cane sugar for cell growth (Hernández et al., 2018). The yeast host holds the GRAS (generally recognized as safe) status and lacks endogenous sucrose- or fructan-transforming enzymes. Immobilization of whole PGFT6x-308 cells retaining *N*-glycosylated *Sa*1-SSTrec in the periplasmic space may result in an invertase-free biocatalyst appropriate for reuse in a cost-effective continuous process that efficiently converts sucrose into 1-kestotriose.

Cell immobilization processes often use insoluble calcium alginate as the entrapping matrix. The method is rapid, nontoxic, inexpensive, versatile, eco-friendly, and recommended for applications in the food and pharmaceutical industries (Smidsrød & Skja, 1990). In bioprocess, Ca-alginate entrapment of enzymes and cells offers relevant advantages, such as greater resistance to external aggressions, increased possibility of enzyme recovery and straightforward enzyme/product separation, reusable biocatalysts with operational stability, and reduction in operating costs (Gonçalves et al., 2019).

Several authors have successfully entrapped enzymes or fungal cells in Ca-alginate gels for continuous FOSs production from sucrose (Cheng et al., 1996; Fernandez-Arrojo et al., 2013; Hayashi et al., 1994; Park et al., 2005; Ganaie et al., 2014; Gonçalves et al., 2020). There are no previous reports on immobilized yeast cells expressing a plant fructosyltransferase. Ca-alginate entrapment of PGFT6x-308 cells may be advantageous over immobilization of the free enzyme. The whole-cell biocatalyst may prevent *Sa*1-SST from leaking out of the beads, but it may cause operational troubles if the confined yeast remains alive and fermentative metabolism of glucose releases the undesired products ethanol, acetate, and CO_2_.

Immobilized microorganisms are often treated with a membrane-permeabilizing agent in order to reduce diffusional barriers and accelerate biocatalytic reactions (He et al., 2015; Panesar et al., 2007, 2011; Park et al., 1994; Yun et al., 1994). Permeabilization treatments using toluene, a hydrophobic solvent, at appropriate levels dramatically decreased the viability of bacteria and yeasts without causing cell lysis (De Smet et al., 1978; Jackson & DeMoss, 1965; Murakami et al., 1980).

In this work, Ca-alginate entrapment of whole *P*. *pastoris* PGFT6x-308 cells was followed by a membrane-permeabilization treatment. The toluene-treated beads showed enhanced specific activity and a dramatic decrease in cell viability. The inactivation of the fermentative metabolism of glucose, a major product of the *Sa*1-SST reaction, in the permeabilized yeast avoided the undesired formation of ethanol, acetate, and CO_2_. The whole-cell biocatalyst was assayed for the production of short-chain FOSs, mainly 1-kestotriose, in repetitive cycles using different sucrose sources.

## Materials and Methods

### Reagents

Sucrose, glucose, fructose, 1-kestotriose, and 1,1-kestotetraose were purchased from Sigma Aldrich (USA). Toluene (≥99.5%) and sodium alginate (source *Laminaria hyperborea*) were purchased from BDH Chemicals (England). Refined sugar, raw sugar, sugarcane syrup, and molasses were collected at the sugar mill “Melanio Hernández,” Tuinucú, Sancti Spíritus, Cuba.

### Strain

*P*. *pastoris* PGFT6x-308 constitutively expressing nine copies of the *1-SST* gene from *S*. *arundinaceus* (Hernández et al., 2018) was maintained on agar plates containing YPG (Yeast extract 10 g/l, peptone 20 g/l, and glucose 20 g/l) with hygromycin (100 μg/ml) at 30°C.

### Culture Conditions

One liter of MM-molasses medium for batch cultures of *P*. *pastoris* PGFT6x-308 contained 22 g NH_4_SO_4_, 18.2 g K_2_HPO_4_, 7.5 g MgSO_4_ 7H_2_O, 0.5 g CaCl_2_ 2H_2_O, 5 g yeast extract, and sugarcane molasses at 5ºBx. Vitamins and trace elements were prepared and used as recommended by d'Anjou and Daugulis (2001). The inoculum was cultured in a 500-ml shake flask with 100 ml of MM-molasses medium using a Minitron shaker (Infors, Switzerland) at 30°C with 250 rpm for 24 hr.

Fed-batch cultures of *P*. *pastoris* PGFT6x-308 were performed in a 5-l fermenter (Infors, Switzerland) with the temperature kept at 30°C and pH 5.5 controlled by the automatic addition of 40% (wt/vol) H_3_PO_4_ and 25% (wt/vol) NH_3_OH. The air flow rate was 3 l/min and the dissolved oxygen was maintained above 20% by increasing automatically the agitation speed from 500 rpm to 900 rpm. After 20 hr of batch cultivation, the air flow rate was adjusted to 6 l/min and the feeding phase was accomplished by the automatic addition of the carbon source at a rate range of 6–8 ml/l/hr according to the carbon demand, as monitored by the level of dissolved oxygen. The feeding solution was sugarcane molasses at 45°Bx. After 72 hr of cultivation, the cells were harvested by centrifugation (4°C, 3000 rpm).

### Cell Immobilization

Whole cells (10, 20, 30, or 40 g, wet biomass) and 2 g of sodium alginate were suspended in water to a final volume of 100 ml and stirred thoroughly (100 rpm) at 25°C to ensure a homogenous distribution before extrusion in the CaCl_2_ solution. The alginate/cell mixtures were dropped through a fine needle into 1 l of 37 mM CaCl_2_ solution with constant stirring (100 rpm) using an impeller, type marine propeller, to avoid droplet aggregation. Gelation time was 1 hr and then the CaCl_2_ solution was discarded. The spherical alginate beads (diameter 2–3 mm) were hardened overnight in 67 mM CaCl_2_ at 4°C and stored in 1.46 M sucrose in 50 mM sodium acetate buffer (pH 5.5) at 4°C before use.

### Permeabilization of Immobilized Cells

Beads (5 g, wet weight) were added to 10 ml of sodium acetate buffer (100 mM, pH 5.5) containing toluene at final concentrations of 3, 5, and 10% (vol/vol). The flasks were shaken at 100 rpm, 30°C for 1 hr to provide adequate contact between the entrapped cells and the solvent floating on the surface of the aqueous solution. After the membrane-permeabilization treatment, beads were recovered by filtration, washed twice with sodium acetate buffer (100 mM, pH 5.5) to remove residual toluene and stored in 1.46 M sucrose in 50 mM sodium acetate buffer (pH 5.5) at 4°C. Beads and solutions were assayed for sucrose-transforming activity.

To determine cell viability, beads were smashed in a liquid YPG medium and aliquots (0.1 ml) of sequential dilutions were plated on YPG agar. The number of colony-forming units (cfu) per gram of wet bead was determined on plates incubated at 30°C for 48 hr. Beads (5 g, wet weight) were incubated in 10 ml of sucrose solution (100 g/l) at 30°C for 12 hr to detect the release of CO_2_ as evidence of cell viability.

### Enzyme Assay

Free or immobilized cells were reacted with 1.75 M sucrose in 100 mM sodium acetate buffer (pH 5.5) in a shaking water bath at 30°C with agitation (50 rpm). After 2 hr, the reaction was stopped by heating 20 min at 80°C. Glucose released to the solution was quantified by the dinitrosalicylic acid (DNSA) colorimetric method (Miller, 1959). One unit of *Sa*1-SSTrec is defined as the amount of enzyme releasing 1 µmol of glucose per minute at initial rates under the reaction conditions described above.

For evaluating the effect of toluene on the stability of free *Sa*1-SSTrec, the enzyme recovered from the culture supernatant of sucrose-grown *P*. *pastoris* PGFT6x-308 (Hernández et al., 2018) was preincubated with the organic solvent (0, 3, 5, and 10% vol/vol) in 100 mM sodium acetate buffer (pH 5.5) at 30°C. The residual *Sa*1-SSTrec activity after 30- and 60-min preincubations was measured with 1.75 M sucrose in 100 mM sodium acetate buffer (pH 5.5).

For assaying pH stability, toluene-treated beads (1 g, wet weight) were preincubated with 60% (wt/vol) sucrose in 100 mM sodium acetate/phosphate buffers of varying pH (4–8) at 30°C. For assaying thermal stability, the entrapped cells (1 g, wet weight) were preincubated with 60% (wt/vol) sucrose in 100 mM sodium acetate buffer (pH 5.5) at temperatures of 30, 40, 50, and 60°C. The residual *Sa*1-SSTrec activity of beads collected at time intervals was measured with 1.75 M sucrose in 100 mM sodium acetate buffer (pH 5.5).

### FOSs Production and Operational Stability Assay

Beads (1 g, wet weight) were incubated in a 10-ml solution of the sucrose source (white refined sugar, raw sugar, sugarcane syrup, or molasses) adjusted to 50°Bx (around 600 g/l) in 100 mM sodium acetate buffer pH 5.5, at 30°C for 48 hr with agitation (100 rpm) in a stirred tank reactor. Samples were collected at regular intervals.

To evaluate the operational stability of the biocatalyst, 15 repetitive batches of 48 hr were performed under the conditions mentioned above. At the end of each cycle, the alginate beads were recovered by filtration and a fresh substrate solution was added. After 30 days, the beads were collected and assayed for *Sa*-1SSTrec activity. The residual activity was established by comparing with the activity of the beads stored in a sucrose solution (1.46 M) at 4°C.

### Carbohydrate Analysis

Qualitative FOSs analysis was performed by thin layer chromatography (TLC) on TLC Silica gel 60 F254 plates (Merck, Germany) using acetone 95% (vol/vol) as a mobile phase. Detection was achieved by spraying with a saturated solution of 1-butanol 80% (vol/vol), urea 3% (wt/vol), H_3_PO_4_ 6% (vol/vol), and absolute ethanol 4% (vol/vol) and heating for 5 min at 120°C.

Quantitative sugar analysis was performed by HPLC using an Aminex HPX-42C column (0.78 × 30 cm, BIORAD) equipped with a refractive index detector. The column temperature was kept at 85°C. Water was used as a mobile phase at a flow rate of 0.5 ml/min. Samples were appropriately diluted before injection. Solutions (20 mg/ml) of fructose, glucose, sucrose, 1-kestotriose, and 1,1-kestotetraose were used as standards. The program package ezData (www.chemilab.net) was used for sugars visualization.

### Statistical Analysis

The statistical package for social sciences (SPSS) 15.0 was used for the data analyses. The data are presented as means ± standard deviation. The level of significance used in this study was *p* < 0.05.

## Results and Discussions

### Performance of Recombinant *P*. *pastoris* PGFT6x-308 in Fed-batch Fermentation With Molasses as Carbon Source

In a previous report, *P*. *pastoris* PGFT6x-308 constitutively expressing nine copies of the *Sa1-SST* was found to secrete the recombinant enzyme (*Sa*1-SSTrec), a fully active glycoprotein, to the cell periplasm and the extracellular medium in fed-batch fermentations using either glycerol or cane sugar as the carbon source (Hernández et al., 2018). The acquired ability of *P. pastoris* PGFT6x-308 to utilize sucrose prompted us to evaluate the use of sugarcane molasses as a cheaper substrate for cell growth.

**Fig. 1.**
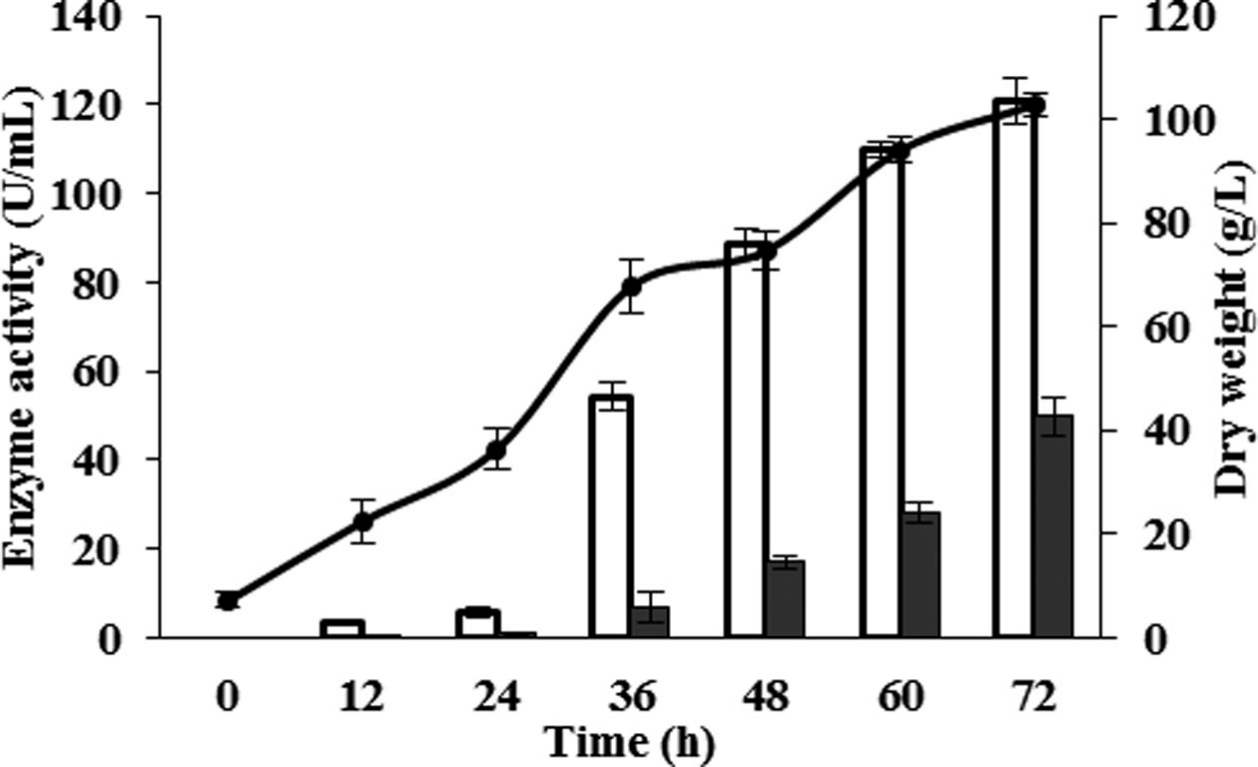
Performance of *Pichia pastoris* PGFT6x-308 in fed-batch fermentation using molasses as a carbon source. Periplasmic (black bars) and extracellular (white bars) *Sa*1-SSTrec activities were measured in intact cells and the culture supernatant, respectively. Biomass yield (line) is expressed as dry weight.

Fig. 1 shows the behavior of *P. pastoris* PGFT6x-308 in a representative fed-batch fermentation using molasses as a carbon source. Cell density and *Sa*1-SSTrec production increased continuously over time (Fig. 1). After 72 hr of culture, *Sa*1-SSTrec activity was detected both in undisrupted biomass (49.8 U/ml of culture, equivalent to 0.48 U/mg of dry cell) and in extracellular medium (120.7 U/ml). The overall *Sa*1-SST activity (170.5 U/ml) was about 5.3- and 1.7-fold higher than the values reported for the carbon sources glycerol and cane sugar, respectively (Hernández et al., 2018). Previous cell fractionation experiments demonstrated that *Sa*1-SSTrec is not attached to membranes but secreted into the periplasmic space, where the substrate sucrose readily enters by diffusion (Hernández et al., 2018). The replacement of glycerol or cane sugar by molasses had no effect on the biomass yield, which remained above 100 g/l (dry weight). The volumetric productivity of *P. pastoris* PGFT6x-308 at the end of cell growth in the molasses-containing medium was 2368.0 U/l/hr.

Reducing sugars (glucose and fructose) and sucrose are the major carbohydrates in sugarcane molasses. Additional glucose is released from sucrose by the action of periplasmic and extracellular *Sa*1-SSTrec during cell growth. The continuous availability of the monosaccharide may have reinforced the transgene transcription. The *GAP* promoter, although constitutive, is known to behave stronger in glucose-grown cells (Waterham et al., 1997; Cereghino & Cregg, 2000).

### Entrapment of *P. pastoris* PGFT6x-308 Cells in Calcium-Alginate Beads

**Fig. 2.**
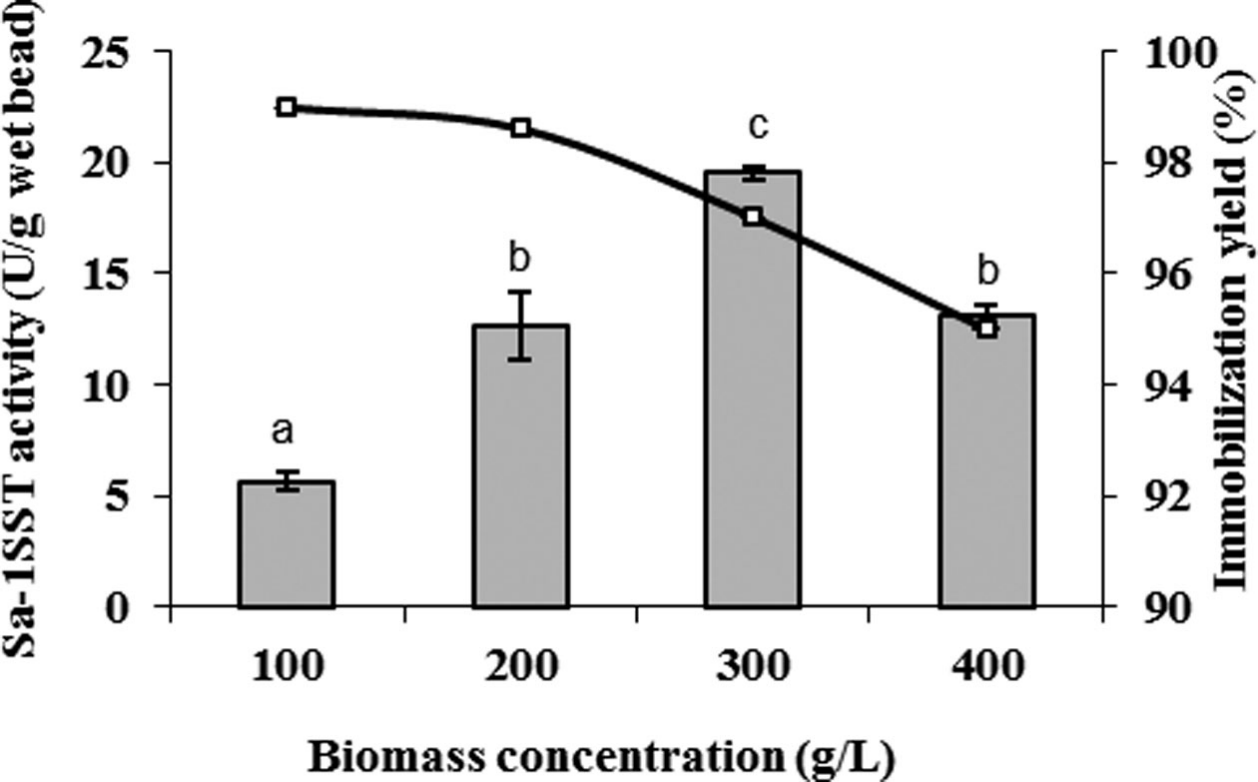
Effect of biomass loading on cell entrapment efficiency (line) and *Sa*1-SSTrec activity (bars). Immobilization yield expresses the percentage of initial *Sa*1-SSTrec activity which was not recovered in the entrapped cells but remained in the CaCl_2_ solution. Data are averages of three experiments with standard deviation. Different letters show significant differences between enzyme activities per bead for each biomass loading (*Student–Newman–Keuls test*, α = 0.05).

Whole cells of *P. pastoris* PGFT6x-308 were entrapped in calcium-alginate gel using different biomass concentrations (100–400 g/l, wet weight) (Fig. 2). The highest recovery of *Sa*-1SSTrec activity was 98.9%, achieved in the beads prepared with the lowest biomass concentration (100 g/l). The immobilization yield decreased to 98, 97, and 95% in the treatment using 200, 300, and 400 g/l of wet biomass, respectively. Similarly, the immobilization yield dropped slightly with the increase in the biomass in the Ca-alginate entrapment process of heat-killed *P. pastoris* cells carrying a recombinant thermostable invertase in the periplasmic space (Martínez et al., 2014). We do not exclude the possibility that in both cases vigorous stirring may have caused some degree of mechanical damage to the cells releasing the soluble periplasmic enzymes to the external medium prior to bead formation.

The inferior *Sa*-1SSTrec activity (5.67 U/g) in the beads formed using the least concentrated biomass suspension (100 g/l) can be attributed to a suboptimal number of entrapped cells. The specific activity of the beads reached the maximum value (19.51 U/g wet bead) when biomass loading was 300 g/l. The treatment with the most concentrated cells (400 g/l) resulted in heavier beads of lower specific activity (13.11 U/g), suggesting a restricted internal diffusion of the substrate sucrose. Similarly, high biomass/alginate ratios failed to increase the activity of Ca-alginate entrapped cells of *Saccharomyces cerevisiae* (Polakovič et al., 2001) or *P*. *pastoris* with the recombinant periplasmic invertase (Martínez et al., 2014).

Scanning electron microscopy (SEM) studies are necessary to confirm the homogenous distribution of whole *P. pastoris* PGFT6x-308 cells inside the alginate spheres (diameter 2–3 mm) as well as to explore the 3D surface structure of the beads. SEM examinations of entrapped whole cells from different microorganisms have allowed a detailed characterization of the internal and external morphology of Ca-alginate beads (Van Neerven et al., 1990; Duarte et al., 2013; Gonçalves et al., 2020).

### Permeabilization of Immobilized *P. pastoris* PGFT6x-308 Cells

**Fig. 3.**
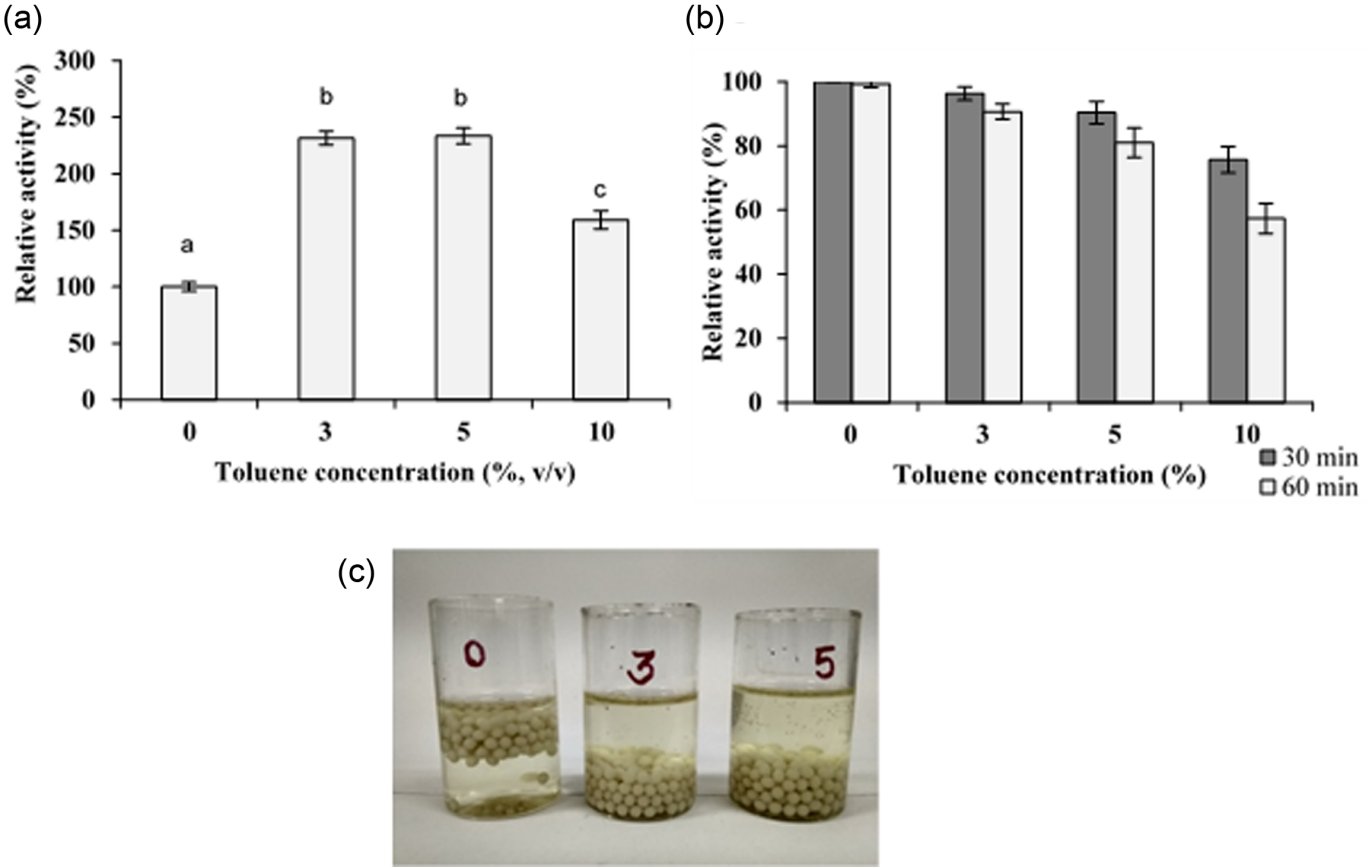
Permeabilization of immobilized *Pichia pastoris* PGFT6x-308 cells. (a) Effect of toluene concentration on the *Sa*1-SSTrec activity of the calcium-alginate beads. (b) Effect of toluene on the stability of free *Sa*1-SSTrec. (c) Reduction of CO_2_ formation by toluene-permeabilized cells during transformation of sucrose (1.75 M) at 30°C. Cut glasses contain beads previously incubated with 0, 3, or 5% (vol/vol) toluene. Data are the means of triplicate measurements ± standard deviation. Different letters are significant differences between relative activity at the three concentrations with respect to the control (*Student–Newman–Keuls test*, α = 0.05).

The spherical Ca-alginate beads (diameter 2–3 mm) with specific sucrase activity of 19.51 U/g were incubated with toluene at three different concentrations. Sucrose conversion by the whole-cell biocatalyst was increased 2.2-, 2.3-, and 1.5-fold after the treatment with 3, 5, and 10% (vol/vol) of toluene, respectively (Fig. 3a). The membrane-permeabilization treatment enhanced the diffusion of the substrate sucrose into the cell increasing the catalytic action of the recombinant enzyme in the periplasmic space. *Sa*1-SSTrec is known to behave more active with the increase of sucrose concentration (Hernández et al., 2018). The activity decay observed when the toluene concentration was raised from 5 to 10% (vol/vol) may be attributed to the enzyme sensibility to the organic solvent. Preincubation of free *Sa*1-SSTrec with toluene in 100 mM sodium acetate buffer (pH 5.5) at 30°C for 1 hr decreased the enzyme stability in direct proportion to the solvent concentration (Fig. 3b). In another experiment, the stirring (100 rpm) of a whole-cell suspension in the presence of 10% (vol/vol) toluene at pH 5.5 and 30°C for 1 hr did not result in the release of *N*-glycosylated periplasmic *Sa*1-SSTrec to the external medium.

Beads that were not exposed to toluene (control beads) produced CO_2_ bubbles and floated up during incubation with 1.75 M sucrose at 30°C. Instead, the toluene-treated beads hardly released gas and sank to the bottom of the flask (Fig. 3c). Cell viability decreased from 10^9^ in the control beads to 10^3^ cfu/g of wet beads after the treatment with toluene at the optimal concentration of 5% (vol/vol). Toluene permeabilization elicited cell death, which is technically advantageous for biocatalyst operation. The reaction of *Sa*1-SSTrec on sucrose releases glucose, which is metabolized by living PGFT6x-308 cells producing the contaminants ethanol and acetate in the FOSs syrup. The glucose fermentation process generates CO_2_. The formation of CO_2_ bubbles by immobilized yeast increases the internal pressure of packed-bed columns which then tend to crack (Najafpour et al., 2004).

Toluene at the assayed concentrations (3, 5, and 10% vol/vol) did not provoke the leakage of periplasmic *Sa*1-SSTrec out of the Ca-alginate matrix, considering that no sucrose-transforming activity was detected in the solutions after beads recovery. We assume that the permeabilization treatment killed the yeast without causing cell disruption. Early microscopic examinations revealed that nonviable bacterial and yeast cells treated with toluene remain essentially intact (Jackson & DeMoss, 1965; De Smet et al., 1978; Murakami et al., 1980).

### Effect of pH and Temperature on *Sa*1-SSTrec Activity and Stability in Immobilized Cells

**Fig. 4.**
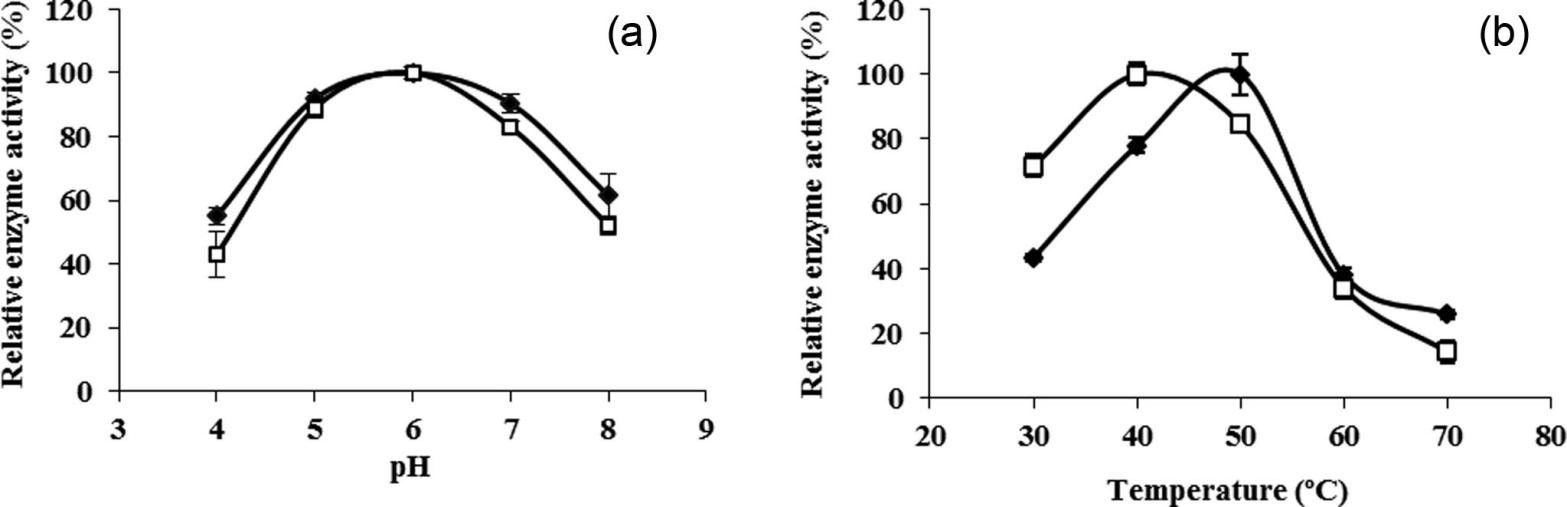
Effect of pH (a) and temperature (b) on the initial rates of *Sa*1-SSTrec activity in free (□) and immobilized (◆) cells. Values are means of three experiments with standard deviation.

The effect of pH on the initial rates of *Sa*1-SSTrec reaction on sucrose (1.75 M) in free and immobilized *P. pastoris* PGFT6x-308 cells was evaluated in the range of 4–8 at 30°C (Fig. 4a). Free cells and toluene-treated beads showed similar pH-activity curves with the highest values at pH 6.0. The use of calcium alginate as the cell entrapment matrix caused no changes in the net charge of the biocatalyst.

The influence of temperature (30–70°C) on the initial rates of *Sa*1-SSTrec reaction on sucrose (1.75 M) in free and immobilized cells was evaluated at pH 5.5 (Fig. 4b). A 10-degree higher optimal temperature (50°C) was observed for the immobilized cells. It is plausible to think that the increase of the reaction temperature reduced the viscosity of the concentrated sucrose solution (1.75 M) enhancing the substrate diffusion into the beads. Similarly, mycelial fructosyltransferases from *Aspergillus flavus* NFCCI 2364 and *Aspergillus oryzae* IPT-301 displayed a higher optimal temperature after cell entrapment in calcium-alginate beads (Ganaie et al., 2014; Gonçalves et al., 2020).

The effect of pH (4–8) and temperature (30–60°C) on *Sa*1-SSTrec stability in nonviable entrapped cells was assayed at a sucrose concentration of 600 g/l to mimic the operational conditions of FOSs synthesis. No significant variations in the enzyme stability were observed for the pH range 5–7 with remaining activities of 97 and 66% after preincubation for 2 and 30 days at 30°C, respectively. The half-life values of *Sa*1-SSTrec in the toluene-treated beads preincubated at 30, 40, and 50°C were 37 days, 8 days, and 18 hr, respectively. The enzyme was rapidly inactivated in the beads exposed to 60°C. The half-life of *Sa*1-SSTrec in the beads at 30°C is comparable to the values reported for immobilized fungal fructosyltransferases, which otherwise are often operated at 50°C due to their higher thermal stability (Chien et al., 2001; Nishizawa et al., 2001; Gonçalves et al., 2020).

### Use of Different Sucrose Sources in FOSs Production by Immobilized Cells

**Fig. 5.**
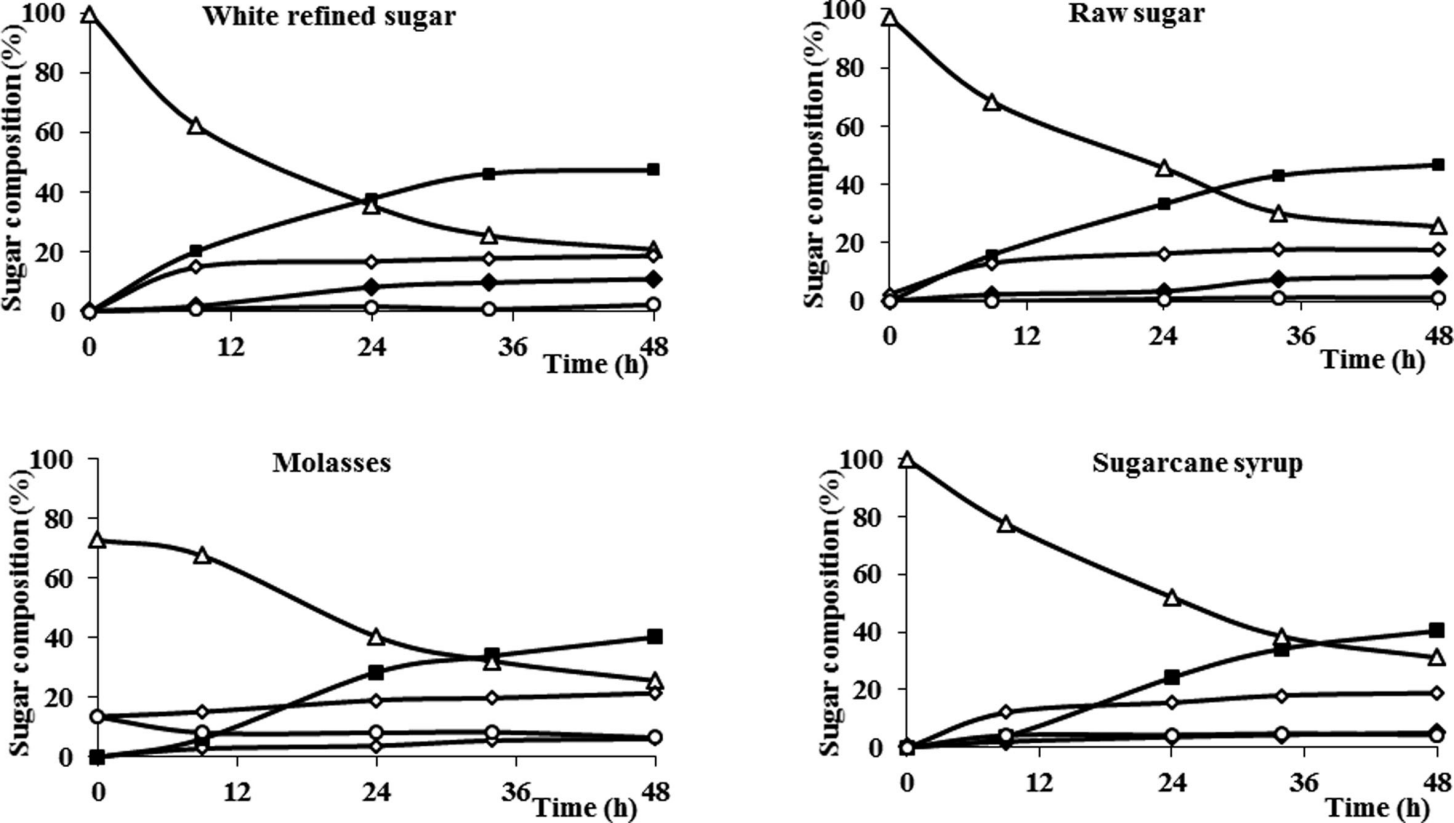
Fructooligosaccharides production by the immobilized whole-cell biocatalyst reacting with different sucrose sources in a stirred tank reactor. Fructose (○), glucose (◊), sucrose (∆), 1-kestotriose (■), and 1,1-kestotetraose (◆). Raw data are included as a supplementary table.

Toluene-treated beads were incubated with refined sugar, raw sugar, sugarcane syrup, and molasses. The reactions were conducted at pH 5.5 and 30°C, and the product composition was monitored for 48 hr (Fig. 5). In all sucrose sources, 1-kestotriose (GF_2_) was the main product, and 1,1-kestotetraose (GF_3_) appeared as the reactions progressed. At the end of the four reactions, the ratio of 1-kestotriose and 1,1-kestotetraose was above 8:2. The overall FOSs yield in the reactions with refined or raw sugar represented above 55% (wt/wt) of total carbohydrates, but it decreased to 45% (wt/wt) with sugarcane syrup and molasses (Table 1). The highest FOSs productivity (7.3 g/l/hr) was achieved when using refined sugar as the substrate. On the other hand, the use of an inexpensive sucrose source as molasses appears particularly attractive for applications that do not require FOSs purification, for instance, animal feed.

**Table 1.** Carbohydrate Composition, Fructooligosaccharide (FOS) Yield and Productivity of the Biocatalyst Reaction With Different Sucrose Sources

Sucrose source	Refined sugar	Raw sugar	Molasses	Sugarcane syrup
	Carbohydrate composition (%, wt/wt)			
Glucose (G)	18.7	17.7	21.4	18.9
Fructose (F)	2.3	1.3	6.6	4.4
Sucrose (GF)	20.8	25.7	25.6	31.1
1-Kestose (GF_2_)	47.3	46.8	40.2	40.4
Nystose (GF_3_)	10.9	8.6	6.2	5.1
**Y_FOS/SUC_** ^a^	58.2	55.4	46.4	45.5
**Y_FOS/BC_** ^b^	3.5	3.3	2.8	2.7
**Y_kestosa/FOS_** ^c^	81.2	84.5	86.7	88.7
**Productivity** ^d^	7.3	6.9	5.7	5.8

^a^Yield was calculated as FOS produced (g)/initial sucrose concentration (g) × 100.

^b^Yield was calculated from FOS produced (g)/biocatalyst (g).

^c^Yield was calculated from 1-kestose produced (g)/FOS produced (g) × 100.

^d^Productivity was calculated from FOS produced (g/l)/time of reaction (hr).

### Reuse of Immobilized Cells in Semicontinuous FOSs Production

**Fig. 6.**
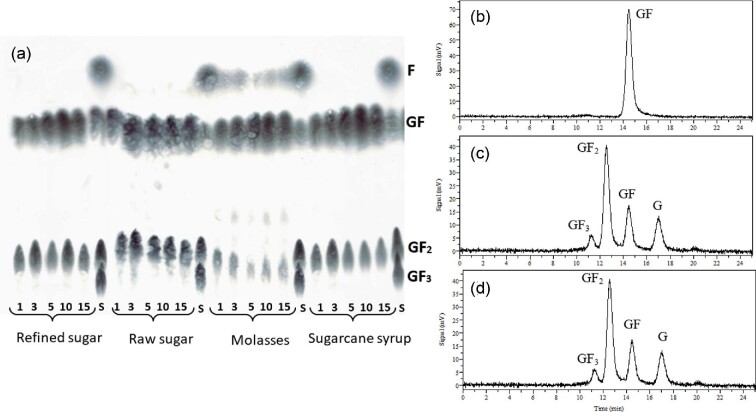
Chromatographic analysis of fructooligosaccharides synthesized by immobilized cells from different sucrose sources in repetitive cycles. (a) TLC chromatograms of samples collected after batches 1, 3, 5, 10, and 15. S means mixture of standard sugars. (b–d) HPLC chromatograms. (b) Refined and raw sugar. (c) Refined sugar after the first batch. (d) Raw sugar after the first batch. GF_3_, 1,1-kestotetraose; GF_2_, 1-kestotriose; GF, sucrose; G, glucose; F, fructose.

Semicontinous FOSs production was performed in stirred tanks at 30°C using refined sugar, raw sugar, sugarcane syrup, and molasses as substrates. The immobilized cells were reused in 15 repeated cycles of 48 hr, each for a total operation time of 30 days. TLC analysis of samples collected after cycles 1, 3, 5, 10, and 15 revealed the synthesis of 1-kestotriose (GF_2_) in the four sucrose sources (Fig. 6). After 5 cycles, the 1-kestotriose yield tended to decrease, particularly when using sugarcane syrup and molasses as the sucrose source.

**Fig. 7.**
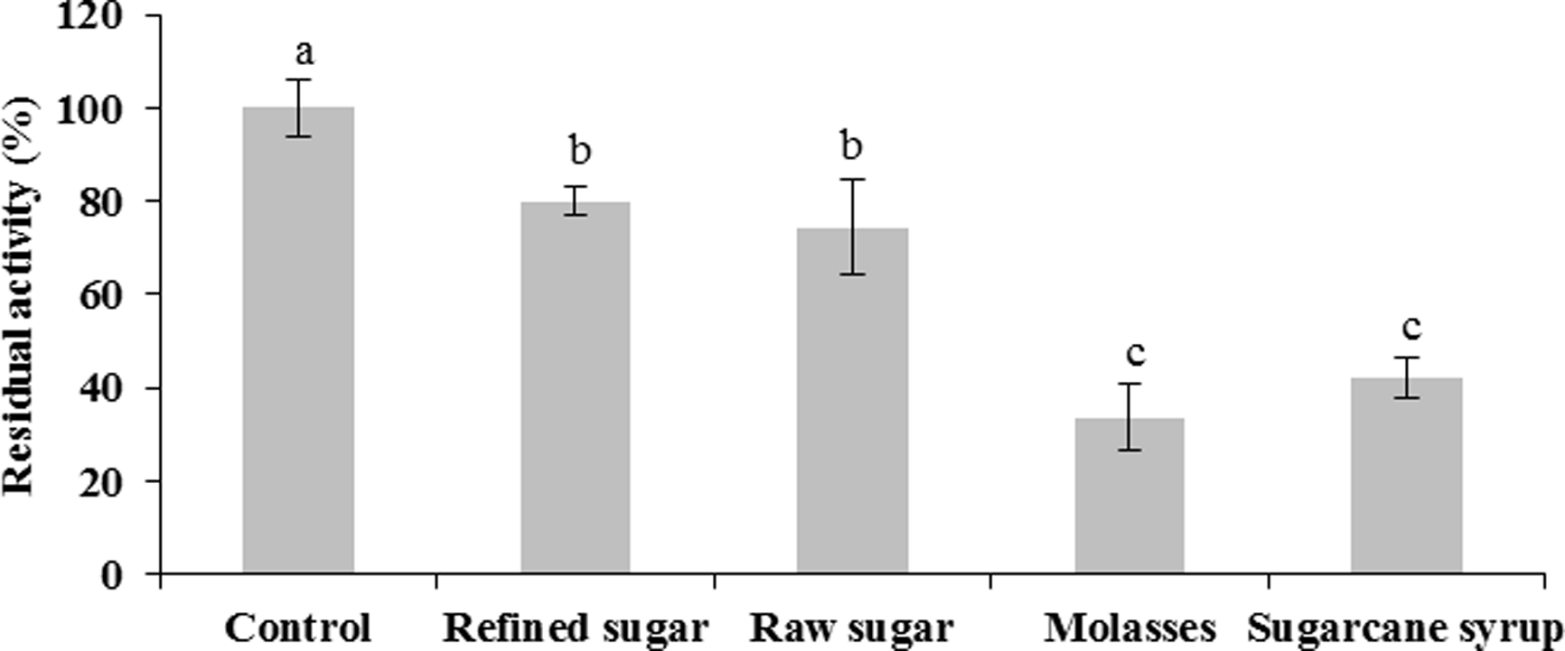
*Sa*1-SSTrec stability in immobilized cells after 15 cycles of batchwise operation. Different letters show significant differences between residual activities in each sucrose source (*Student–Newman–Keuls test*, α = 0.05).

The stability of the biocatalyst was evaluated at the end of the 30-day batchwise operation at 30°C (Fig. 7). The residual activity of the beads incubated with sugarcane syrup and molasses was 41 and 32%, respectively. The continuous exposition with molasses softened and deformed the beads. Deterioration of the alginate matrix might have been caused by cations as manganese and zinc, with lower stabilizing power than calcium, as well as chelating agents as phosphates (Thu et al., 1996).

More attractively, the beads reacted with refined or raw sugar retained 75–80% of the initial *Sa*1-SSTrec activity. The high enzyme stability in the immobilized biocatalyst allows producing repetitive FOSs batches and offers the possibility to increase the residence time in the last cycles during prolonged reuse.

To our knowledge, this work is the first report of immobilized yeast cells expressing a plant fructosyltranferase. While *Sa*1-SSTrec recovered from the culture supernatant (free enzyme) of *P. pastoris* PGFT6x-308 is appropriate for sucrose conversion into 1-kestotriose in one-cycle batch reactions (Hernández et al., 2018), the stable performance of periplasmic *Sa*1-SSTrec in Ca-alginate entrapped cells during repetitive operation cycles makes the immobilized whole-cell biocatalyst attractive for continuous FOSs production. The reduction of diffusional barriers in the toluene-treated beads not only accelerated the *Sa*1-SSTrec reaction but also induced cell death avoiding the formation of undesired metabolic by-products (ethanol, acetate, and CO_2_) during sucrose transformation. A heating treatment killed *P. pastoris* expressing a bacterial thermophilic invertase and enhanced sucrose hydrolysis in the cell periplasm (Martínez et al., 2014). Here, we show that membrane permeabilization is a valid option for the operational optimization of immobilized yeast cells expressing a mesophilic fructosyltransferase specialized in 1-kestotriose synthesis.

## Conclusions

*P*. *pastoris* PGFT6x-308 cells constitutively expressing *1-SST* from *S*. *arundinaceus* (*Sa*) were entrapped in calcium-alginate beads and exposed to a membrane-permeabilizing agent. In the toluene-treated beads, the enhancement of substrate and products diffusion through the outer membrane increased the catalytic action of periplasmic *Sa*1-SSTrec. The immobilized whole-cell biocatalyst yielded 55% (wt/wt) FOSs, 1-kestotriose, and 1,1-kestotetraose in a ratio above 8:2, when reacted with refined or raw sugar (600 g/l) in a stirred tank reactor at 30°C and pH 5.5. The beads retained 75–80% of the initial *Sa*1-SSTrec activity after 15 repetitive cycles of FOSs production in a 30-day batchwise operation. The immobilized biocatalyst is suitable for reuse and cost-effective production of 1-kestotriose, the most attractive commercial FOS.

## Supplementary Material

kuab036_Supplemental_File

## Data Availability

Experimental data are provided in the manuscript. Authors agree to provide any other data if requested.

## References

[bib1] Cereghino J. L., Cregg J. M. (2000). Heterologous protein expression in the methylotrophic yeast *Pichia pastoris*. FEMS Microbiology Reviews, 24(1), 45–66. 10640598 10.1111/j.1574-6976.2000.tb00532.x

[bib2] Cheng C. Y., Duan K. J., Sheu D. C., Lin C. T., Li S. Y. (1996). Production of fructooligosaccharides by immobilized mycelium of *Aspergillus japonicus*. Journal of Chemical Technology & Biotechnology, 66(2), 135–138.

[bib3] Chien C. S., Lee W. C., Lin T. J. (2001). Immobilization of *Aspergillus japonicus* by entrapping cells in gluten for production of fructooligosaccharides. Enzyme and Microbial Technology, 29(4-5), 252–257.

[bib4] Cunha J. S., Ottoni C. A., Morales S. A. V., Silva E. S., Maiorano A. E., Perna R. F. (2019). Synthesis and characterization of fructosyltransferase from *Aspergillus oryzae* IPT-301 for high fructooligosaccharides production. Brazilian Journal of Chemical Engineering, 36(2), 657–668.

[bib5] d'Anjou M. C., Daugulis A. J. (2001). A rational approach to improving productivity in recombinant *Pichia pastoris* fermentation. Biotechnology and Bioengineering, 72(1), 1–11. 11084587 10.1002/1097-0290(20010105)72:1<1::aid-bit1>3.0.co;2-t

[bib6] De Smet M. J., Kingma J., Witholt B. (1978). The effect of toluene on the structure and permeability of the outer and cytoplasmic membranes of *Escherichia coli*. Biochimica et Biophysica Acta (BBA) - Biomembranes, 506(1), 64–80. 413578 10.1016/0005-2736(78)90435-2

[bib7] Duarte J. C., Rodrigues J. A. R., Moran P. J. S., Valença G. P., Nunhez J. R. (2013). Effect of immobilized cells in calcium alginate beads in alcoholic fermentation. AMB Express, 3(1), 31. 23721664 10.1186/2191-0855-3-31 PMC3695878

[bib8] Faria L. L., Morales S. A. V., Prado J. P. Z., Dias G. S., de Almeida A. F., Xavier M. C. A., da Silva E. S., Maiorano A. E., Perna R. F. (2021). Biochemical characterization of extracellular fructosyltransferase from *Aspergillus oryzae* IPT-301 immobilized on silica gel for the production of fructooligosaccharides. Biotechnology Letters, 43(1), 43–59. 33025334 10.1007/s10529-020-03016-7

[bib9] Fernandez R. C., Ottoni C. A., da Silva E. S., Matsubara R. M. S., Carter J. M., Magossi L. R., Wada M. A. A., de Andrade Rodrigues M. F., Maresma B. G., Maiorano A. E. (2007). Screening of β-fructofuranosidase-producing microorganisms and effect of pH and temperature on enzymatic rate. Applied Microbiology and Biotechnology, 75(1), 87–93. 17375295 10.1007/s00253-006-0803-x

[bib10] Fernandez-Arrojo L., Rodriguez-Colinas B., Gutierrez-Alonso P., Fernandez-Lobato M., Alcalde M., Ballesteros A. O., Plou F. J. (2013). Dried alginate-entrapped enzymes (DALGEEs) and their application to the production of fructooligosaccharides. Process Biochemistry, 48(4), 677–682.

[bib11] Ganaie M. A., Rawat H. K., Wani O. A., Gupta U. S., Kango N. (2014). Immobilization of fructosyltransferase by chitosan and alginate for efficient production of fructooligosaccharides. Process Biochemistry, 49(5), 840–844.

[bib12] Gonçalves M. C. P., Kieckbusch T. G., Perna R. F., Fujimoto J. T., Morales S. A. V., Romanelli J. P. (2019). Trends on enzyme immobilization researches based on bibliometric analysis. Process Biochemistry, 76, 95–110.

[bib13] Gonçalves M. C. P., Morales S. A. V., Silva E. S., Maiorano A. E., Perna R. F., Kieckbusch T. G. (2020). Entrapment of glutaraldehyde crosslinked cells from *Aspergillus oryzae* IPT-301 in calcium alginate for high transfructosylation activity. Journal of Chemical Technology & Biotechnology, 95(9), 2473–2482.

[bib14] Hayashi S., Tubouchi Y., Takasaki Y., Imada K. (1994). Long-term continuous reaction of immobilized β-fructofuranosidase. Biotechnology Letters, 16(3), 227–228.

[bib15] He Y. C., Liu F., Zhang D. P., Gao S., Li Z. Q., Tao Z. C., Ma C. L. (2015). Biotransformation of 1,3-propanediol cyclic sulfate and its derivatives to diols by toluene-permeabilized cells of *Bacillus* sp. cczu11-1. Applied Biochemistry and Biotechnology, 175(5), 2647–2658. 25547816 10.1007/s12010-014-1457-2

[bib16] Hernández L., Menéndez C., Pérez E. R., Martínez D., Alfonso D., Trujillo L. E., Ramírez R., Sobrino A., Mazola Y., Musacchio A., Pimentel E. (2018). Fructooligosaccharides production by *S*c*hedonorus arundinaceus* sucrose:sucrose 1-fructosyltransferase constitutively expressed to high levels in *Pichia pastoris*. Journal of Biotechnology, 266, 59–71. 29246839 10.1016/j.jbiotec.2017.12.008

[bib17] Jackson R. W., DeMoss J. A. (1965). Effects of toluene on *Escherichia coli*. Journal of Bacteriology, 90(5), 1420–1425. 5321488 10.1128/jb.90.5.1420-1425.1965 PMC315830

[bib18] Kaplan H., Hutkins R. W. (2000). Fermentation of fructooligosaccharides by lactic acid bacteria and bifidobacteria. Applied and Environmental Microbiology, 66(6), 2682–2684. 10831458 10.1128/aem.66.6.2682-2684.2000 PMC110601

[bib19] Mabel M. J., Sangeetha P. T., Platel K., Srinivasan K., Prapulla S. G. (2008). Physicochemical characterization of fructooligosaccharides and evaluation of their suitability as a potential sweetener for diabetics. Carbohydrate Research, 343(1), 56–66. 18005951 10.1016/j.carres.2007.10.012

[bib20] Maiorano A. E., da Silva E. S., Perna R. F., Ottoni C. A., Piccoli R. A. M., Fernandez R. C., Maresma B. G., de Andrade Rodrigues M. F. (2020). Effect of agitation speed and aeration rate on fructosyltransferase production of *Aspergillus oryzae* IPT-301 in stirred tank bioreactor. Biotechnology Letters, 42(12), 2619–2629. 32979133 10.1007/s10529-020-03006-9

[bib21] Martínez D., Menéndez C., Echemendia F. M., Pérez E. R., Trujillo L. E., Sobrino A., Ramírez R., Quintero Y., Hernández L. (2014). Complete sucrose hydrolysis by heat-killed recombinant *Pichia pastoris* cells entrapped in calcium alginate. Microbial Cell Factories, 13(1), 87–95. 24943124 10.1186/1475-2859-13-87 PMC4078364

[bib22] Mendlik K., Albrecht J. A., Schnepf M. (2012). Effects of fructooligofructoses chain length on the bifidobacteria of the human colon: A pilot study. Food and Nutrition Sciences, 3, 1615–1618.

[bib23] Miller G. L. (1959). Use of dinitrosalicylic acid reagent for determination of reducing sugar. Analytical Chemistry, 31(3), 426–428.

[bib24] Murakami K., Nagura H., Yoshino M. (1980). Permeabilization of yeast cells: Application to study on the regulation of AMP deaminase activity *in situ*. Analytical Biochemistry, 105(1), 407–413. 7006447 10.1016/0003-2697(80)90479-0

[bib25] Najafpour G., Younesi H., Ismail K. S. K. (2004). Ethanol fermentation in an immobilized cell reactor using *Saccharomyces cerevisiae*. Bioresource Technology, 92(3), 251–260. 14766158 10.1016/j.biortech.2003.09.009

[bib26] Nishizawa K., Nakajima M., Nabetani H. (2001). Kinetic study on transfructosylation by β-fructofuranosidase from *Aspergillus niger* ATCC 20611 and availability of a membrane reactor for fructooligosaccharide production. Food Science and Technology Research, 7, 39–44.

[bib27] Panesar P. S., Panesar R., Singh R. S., Bera M. B. (2007). Permeabilization of yeast cells with organic solvents for β-galactosidase activity. Research Journal of Microbiology, 2, 34–41.

[bib28] Panesar R., Panesar P. S., Singh R. S., Kennedy J. F. (2011). Hydrolysis of milk lactose in a packed bed reactor system using immobilized yeast cells. Journal of Chemical Technology and Biotechnology, 86(1), 42–46.

[bib29] Park M. C., Lim J. S., Kim J. C., Park S. W., Kim S. W. (2005). Continuous production of neo-fructooligosaccharides by immobilization of whole cells of *Penicillium citrinum*. Biotechnology Letters, 27(2), 127–130. 15703876 10.1007/s10529-004-7339-x

[bib30] Park Y. M., Choi E. S., Rhee S. K. (1994). Effect of toluene-permeabilization on oxidation of D-sorbitol to L-sorbose by *Gluconobacter suboxydans* cells immobilized in calcium alginate. Biotechnology Letters, 16(4), 345–348.

[bib31] Pereira I. A., Gibson G. R. (2002). Effects of consumption of probiotics and prebiotics on serum lipid levels in humans. Critical Reviews in Biochemistry and Molecular Biology, 37(4), 259–281. 12236466 10.1080/10409230290771519

[bib32] Perna R. F., Cunha J. S., Gonçalves M. C. P., Basso R. C., Silva E. S., Maiorano A. E. (2018). Microbial fructosyltransferase: Production by submerged fermentation and evaluation of pH and temperature effects on transfructosylation and hydrolytic enzymatic activities. International Journal of Engineering Research and Science, 4(3), 43–50.

[bib33] Polakovič M., Kudláčová G., Štefuca V., Báleš V. (2001). Determination of sucrose effective diffusivity and intrinsic rate constant of hydrolysis catalysed by Ca-alginate entrapped cells. Chemical Engineering Science, 56(2), 459–466.

[bib34] Rehm J., Willmitzer L., Heyer A. G. (1998). Production of 1-kestose in transgenic yeast expressing a fructosyltransferase from *Aspergillus foetidus*. Journal of Bacteriology, 180(5), 1305–1310. 9495772 10.1128/jb.180.5.1305-1310.1998 PMC107021

[bib35] Sánchez-Martínez M. J., Soto-Jover S., Antolinos V., Martínez-Hernández G. B., López-Gómez A. (2020). Manufacturing of short-chain fructooligosaccharides: From laboratory to industrial scale. Food Engineering Reviews, 12(2), 149–172.

[bib36] Smidsrød O., Skja G. (1990). Alginate as immobilization matrix for cells. Trends in Biotechnology, 8, 71–78. 1366500 10.1016/0167-7799(90)90139-o

[bib37] Suzuki N., Aiba Y., Takeda H., Fukumori Y., Koga Y. (2006). Superiority of 1-kestose, the smallest fructo-oligosaccharide, to a synthetic mixture of fructo-oligosaccharides in the selective stimulating activity on bifidobacteria. Bioscience and Microflora, 25(3), 109–116.

[bib38] Thu B., Bruheim P., Espevik T., Smidsrød O., Soon-Shiong P., Skjåk-Braek G. (1996). Alginate polycation microcapsules: I. Interaction between alginate and polycation. Biomaterials, 17(10), 1031–1040. 8736740 10.1016/0142-9612(96)84680-1

[bib39] Trujillo L. E., Arrieta J. G., Dafhnis F., García J., Tambara Y., Pérez M., Hernández L. (2001). Fructo-oligosaccharides production by the *Gluconacetobacter diazotrophicus* levansucrase expressed in the methilotrophic yeast *Pichia pastoris*. Enzyme and Microbial Technology, 28(2-3), 139–144. 11166804 10.1016/s0141-0229(00)00290-8

[bib40] Van Neerven A. R. W., Wijffels R. H., Zehnder A. J. B. (1990). Scanning electron microscopy of immobilized bacteria in gel beads: A comparative study of fixation methods. Journal of Microbiological Methods, 11(3-4), July 1990, 157–168.

[bib41] Waterham H. R., Digan M. E., Koutz P. J., Lair S. V., Cregg J. M. (1997). Isolation of the *Pichia pastoris* glyceraldehyde-3-phosphate dehydrogenase gene and regulation and use of its promoter. Gene, 186(1), 37–44. 9047342 10.1016/s0378-1119(96)00675-0

[bib42] Yang H., Wang Y., Zhang L., Shen W. (2016). Heterologous expression and enzymatic characterization of fructosyltransferase from *Aspergillus niger* in *Pichia pastoris*. New Biotechnology, 33(1), 164–170. 25976629 10.1016/j.nbt.2015.04.005

[bib43] Yun J. W., Lee M. G., Song S. K. (1994). Continuous production of isomalto-oligosaccharides from maltose syrup by immobilized cells of permeabilized *Aureobasidium pullulans*. Biotechnology Letters, 16(11), 1145–1150.

