# Supplementary material for: Fructooligosaccharides production by immobilized Pichia pastoris cells expressing Schedonorus arundinaceus sucrose:sucrose 1-fructosyltransferase
Source: J Ind Microbiol Biotechnol. 2021 Jun 17;48(5-6):kuab036. doi: 10.1093/jimb/kuab036 (PMC9113426; doi:10.1093/jimb/kuab036)
Supplement: kuab036_Supplemental_File [file kuab036_Supplemental_File.docx]

| Raw data of carbohydrate composition (g/L) from the biocatalyst reaction with different sucrose sources | | | | | |
| --- | --- | --- | --- | --- | --- |
|  |  | **Refined sugar** | **Row sugar** | **Sugar cane syrup** | **Molasses** |
|  | Glucose (G) | 88.8 | 79.2 | 73.2 | 91.2 |
|  | Fructose (F) | 5.4 | 0.6 | 25.8 | 49.2 |
| 9 h | Sucrose (GF) | 374.4 | 410.4 | 465.0 | 405.0 |
|  | Nystose (GF_3_) | 10.8 | 14.4 | 12.6 | 17.4 |
|  | 1-Kestose (GF_2_) | 120.6 | 96.0 | 24.0 | 37.2 |
|  | Glucose (G) | 100.8 | 98.4 | 93.6 | 114.6 |
|  | Fructose (F) | 9.6 | 5.4 | 26.4 | 49.2 |
| 24 h | Sucrose (GF) | 213.0 | 274.2 | 312.6 | 242.4 |
|  | Nystose (GF_3_) | 49.2 | 21.0 | 22.2 | 22.8 |
|  | 1-Kestose (GF_2_) | 226.8 | 200.4 | 145.8 | 171.0 |
|  | Glucose (G) | 106.8 | 103.8 | 108.6 | 119.4 |
|  | Fructose (F) | 4.8 | 7.8 | 29.4 | 50.4 |
| 34 h | Sucrose (GF) | 152.4 | 181.8 | 231.0 | 193.2 |
|  | Nystose (GF_3_) | 58.2 | 45.0 | 28.8 | 33.6 |
|  | 1-Kestose (GF_2_) | 277.2 | 258.6 | 231.0 | 193.2 |
|  | Glucose (G) | 112.2 | 106.2 | 113.4 | 128.4 |
|  | Fructose (F) | 13.8 | 7.8 | 26.4 | 39.6 |
| 48 h | Sucrose (GF) | 124.8 | 154.2 | 186.6 | 153.6 |
|  | Nystose (GF_3_) | 65.4 | 51.6 | 30.6 | 37.2 |
|  | 1-Kestose (GF_2_) | 283.8 | 280.8 | 242.4 | 241.2 |
